# Inferring and analysis of social networks using RFID check-in data in China

**DOI:** 10.1371/journal.pone.0178492

**Published:** 2017-06-01

**Authors:** Tao Liu, Lintao Yang, Shouyin Liu, Shuangkui Ge

**Affiliations:** 1 College of Physical Science and Technology, Central China Normal University, Wuhan, Hubei Province, China; 2 School of Information Science and Technology, Jiujiang University, Jiujiang, Jiangxi Province, China; 3 Collaborative & Innovative Center for Educational Technology, Central China Normal University, Wuhan, Hubei Province, China; 4 Beijing Institute of Electronics Technology and Application, Beijing, China; Centre de physique theorique, FRANCE

## Abstract

Social networks play an important role in our daily lives. However, social ties are rather elusive to quantify, especially for large groups of subjects over prolonged periods of time. In this work, we first propose a methodology for extracting social ties from long spatio-temporal data streams, where the subjects are 17,795 undergraduates from a university of China and the data streams are the 9,147,106 time-stamped RFID check-in records left behind by them during one academic year. By several metrics mentioned below, we then analyze the structure of the social network. Our results center around three main observations. First, we characterize the global structure of the network, and we confirm the small-world phenomenon on a global scale. Second, we find that the network shows clear community structure. And we observe that younger students at lower levels tend to form large communities, while students at higher levels mostly form smaller communities. Third, we characterize the assortativity patterns by studying the basic demographic and network properties of users. We observe clear degree assortativity on a global scale. Furthermore, we find a strong effect of grade and school on tie formation preference, but we do not find any strong region homophily. Our research may help us to elucidate the structural characteristics and the preference of the formation of social ties in college students’ social network.

## Introduction

Social networks play an important role in our daily lives [1, 2]. They enable us to connect, communicate, and share information based on our social connections such as friendship, work-associations, or others [3]. Due to the omnipresence of social networks in our lives, the power of social ties has been leveraged for several applications [4–6]. Hence, effective identification of these social ties can lead to better understanding of the users. Social network analysis, through interviews, surveys and questionnaires can reveal social ties within a small group [7]. However, for large diverse groups, and over prolonged periods of time, the information about social ties is rather difficult to collect. Recent advancements in modern information and communication technologies using social sensors, such as smartphones, the internet, Wi-Fi, and radio frequency identification (RFID) devices, have opened up new ways for organizations to intelligently collect electronic data traces across a variety of communication channels and very different spatial and temporal scales [8]. These data traces can offer unparalleled opportunities to investigate the interplay of real-life social networks, human mobility and dynamical processes.

Researchers have experimented with social traces and connections to discover innovative insights regarding our social lives. Kossinets et al. [9] analyzed e-mail logs from 43,553 undergraduate and graduate students, faculty, and staff members of a large university over the course of one academic year, and showed the pattern of social ties as a function of existing social structure, shared activities among the people, and personal attributes. An analysis of mobile phone call records has demonstrated a local coupling between the tie strength and network topology [10]. The study of social networks in a dynamic perspective has been ongoing over the past few decades. Starting with a series of landmark studies, we found that human societies and communities have complex social dynamics. [11, 12]. Even though such analysis furthered our understanding of the webs of social ties profoundly, important questions have remained unanswered. Researchers have debated whether electronic data offers new methods of creating intimacy or are inflated measures of social connectedness that skim the surface of real attachments [13]. Therefore, some other studies have focused on the problem of inferring social ties from electronic data. Eagle et al. [14] investigated how friendships can be inferred from users’ geo-localized information through Wi-Fi, GPS and Bluetooth logs. They found that dyadic friends demonstrate distinctive temporal and spatial patterns in their physical proximity and calling patterns. Crandall et al. [15] studied an online photo-sharing community, Flickr, and discovered that the presence of even a very small number of spatio-temporal co-located images can indicate a high empirical likelihood of a social tie. Tang W et al. [16] investigated the extent to which social network analysis can yield the types of existing social ties. Sapiezynski et al. [17, 18] showed that it was possible to accurately infer person-to-person physical proximity and to implement high-resolution outdoor positioning from the lists of WiFi access points. Tang et al. [19] demonstrated that social theories (such as structural balance, structural hole, and social status) can be exploited to study, infer and generalize the presence of social ties across multiple heterogeneous social networks.

Recently, the statistical validation method has also been used for the inferring of social ties in diverse domains such as biological networks [20], financial markets [21], and mobile phone networks [22, 23] among others. In this paper, we propose an unsupervised method based on statistical validation for inferring social ties from RFID check-in data of 17,795 undergraduate students from a university in China. Similar to the approach presented in the works of Crandall et al. [15], we use spatio-temporal co-occurrence as a measure of similarity or relationship between the users (students). In our case, the spatio-temporal co-occurrence between two students means that they visited the same location within a short time period. the links are drawn between students based on their co-occurrences. Then we use the method, which has been used in the co-occurences of words in sentences [24], to compute the p-value and validate the presence of a link. Therefore, our methodology can be regarded as a combination of the two existing techniques. We use “co-occurrence” as a short form for “spatio-temporal co-occurrence” unless specifically stated otherwise. The naive approach is to use the raw counts of co-occurrences between students as weighted links. The problem of this approach is that it is encumbered with noise links which are produced by co-occurrences that may happen due to coincidence. Such coincidences are frequent in settings where there are natural peak-hours of similar activity in the target population during the data collection period. To address this problem, we use our method to distinguish between links that denote social ties and the ones that result due to coincidence, generating statistically validated co-occurrence network (SVCN) and verifying this network through 46 subjects.

We use a wide variety of network measures to mine information from the SVCN. We characterize the global structure of the network and validate the small-world phenomenon already observed in real-world and artificial networks. We also analyze the degree distribution and community structure property. Furthermore, we study the degree assortativity and attribute assortativity on global and local scales respectively. We reveal some phenomena: the larger community gradually split into smaller ones with the growth of the grade; the distribution of the number of the students’ social proximity ties is significantly different; it is quite easier to form social proximity ties intra-grade and intra-school.

## Materials and methods

### Ethics statement

This study complies with the guidelines of the 1964 Declaration of Helsinki. The Institutional Review Board (IRB) from Central China Normal University approved the study. However, the study did not consider any clinical tests to be performed for the subjects. In fact it was based on 17,795 undergraduates’ check-in data in student canteens and stores, and information specifying a range of students’ attributes. But there is still a thorny problem need to be solved: how can we ensure that every verbal informed consent be given such a huge number of students. Fortunately, in most of the universities in China, each class is usually equipped with a tutor. For this reason, all subjects included in this study gave oral informed consent through the tutors. In addition, to protect the privacy of the students, all the data was anonymized by encrypting the student IDs. All subsequent computations were performed on this anonymized data.

### Data description

We collected the check-in data from the Student Card System (SCS), at the Central China Normal University, Hubei, China. The system includes three components, which are RFID tags system, student card and the database. Once a student enrolled, he/ she would be assigned a unique student number. Each student will also have a unique card barcoded with student number. This so called “Student Card” functions as ID card, stored-value card and consumer card on campus. RFID tags are armed in several locations such as student canteens, dormitories, library, classrooms and stores across the university campuses. Generally, almost every campus activity obtains a student card check-in. For instance, a student wants to enter the library, his card should be scanned, thus this library check-in data, which includes the information of student number, location (library) and time record, would upload to the database. In this way, the university database has recorded most of the student campus activities.

The work described here is a part of data we extracted from SCS database, including activities occurred in student canteens and stores. When students go to the student canteens for dinner, they should have their card scanned to pay for the meal. Since the RFID tags are armed in 10 student canteens and 7 stores all around the university campuses (as is shown in S1 Fig), we extracted 9,147,106 pieces of data from 17,795 students containing the freshman, sophomore, junior and senior during one academic year (from September, 2015 to June, 2016). Each entry of the records contains the following information: *student ID*, *location* (canteen’s or store’s number) and *timestamp* (the time that the activity occurred). Full details on data collection and data format description are available in S1 Text. Considering that students spend the majority of time on campus, their consuming activities can in a certain conclude.

After we have collected these data, we also acquire the student basic information in another database though student number, such as gender, age, grade, school and region.

### Statistical validation co-occurrences network

Let the check-in data *D* be represented in the form $D={\left\{s_{z},t_{z},l_{z}\right\}}_{z=1}^{Z}$, where *Z* is the total number of records. Each record {*s*_*z*_, *t*_*z*_, *l*_*z*_}, can be read as “the student *s*_*z*_ appeared at time *t*_*z*_ at the location *l*_*z*_”. Note that *s*_*z*_ ∈ {1, …, *N*} denotes the index of the student, *l*_*z*_ ∈ {1, …, *L*} denotes the index of the location, and *t*_*z*_ denotes the timestamp when the observation *z* took place. For a specific student *i*, there can be many records of *z* for which *s*_*z*_ = *i*, as a single individual may appear several times in our check-in dataset. Our goal is to find an appropriate mapping from the check-in data *D* to a student’s social network described by *A* ∈ *R*^*N*×*N*^, where *a*_*ij*_ = 1 denotes the presence of a link between two students *i* and *j*, and *a*_*ij*_ = 0 denotes the absence of such a link.

To do this, we first pre-process our check-in data *D*. Our main assumption is that if two people visit the same location within a short time, they may have some form of social affiliation. We define this spatio-temporal co-occurrence of two students as an instance in which they both appear at the same place, at approximately the same time. Specifically, the entire time is split into discrete intervals, i.e., we only consider integral multiples of a fixed timeslot interval *τ* (for example, *τ* = 5min). We segment our check-in data $D={\left\{s_{z},t_{z},l_{z}\right\}}_{z=1}^{Z}$ into *K* segments. A segment is defined as a sequence of records which contains the check-ins of all the users at the same location within the same fixed timeslot. We say that two students co-occur (or have a co-occurrence) if they are both present in the same segment. As there is a many-to-one correspondence between a given student *i* and segments *k* (e.g. a single student can occur many times in the same segment), we compress multiple such occurrences into a single occurrence.

Here the projection procedure is done by validating each link of the student’s social network *A* against a null hypothesis that a student’s appearance in a certain segment is random. The details of the test are as follows. For two students *i* and *j*, there are two metrics: *n*_*i*_ is the number of segments where the student *i* occurs, *n*_*j*_ is the number of segments where the student *j* occurs. Let *X*_*ij*_ be the number of co-occurrences, i.e., the number of segments where both students *i* and *j* occur. Under this null hypothesis, the test assigns a p-value *p*(*X*_*ij*_) to the link (*i*, *j*). The p-value *p*(*X*_*ij*_) associated with the link (*i*, *j*) is the probability that at least an *X*_*ij*_ number of co-occurrences occurred. We then compare the p-value *p*(*X*_*ij*_) with a statistical threshold. When the p-value *p*(*X*_*ij*_) less than this statistical threshold, the null hypothesis is disproved. Hence, the co-occurrences are not random or due to chance alone. Thus, we can assume that the students *i* and *j* have a social tie.

The p-value *p*(*X*_*ij*_) is obtained as follows. Let *I*_*pqk*_ be an indicator variable which has a value is one if the *p*:th occurrence of the student *i* and *q*:th occurrence of the student *j* are both found in segment *k*, and zero otherwise. The expected number of co-occurrences can be written as the sum of *Kn*_*i*_ *n*_*j*_ terms as follows:

$$\begin{array}{c} \;E\left(X_{ij}\right)=E\left(\sum_{p=1}^{n_{i}}\sum_{q=1}^{n_{j}}\sum_{k=1}^{K}I_{pqk}\right)=\sum_{p=1}^{n_{i}}\sum_{q=1}^{n_{j}}\sum_{k=1}^{K}E\left(I_{pqk}\right)\; \end{array}$$ (1)

Note that the expected value of an indicator variable *E*(*I*_*pqk*_) is the probability of the event occurring. Under the null hypothesis of random co-occurrences, the probability that the student *i*’ *p*:th occurrence in the segment *k* is $\frac{L_{k}}{M}$, where *L*_*k*_ is the size of the segment *k* (i.e., the number of students in the segment *k*), and $M=\sum_{k=1}^{K}L_{k}$. Given that the student *i* is present in the segment *k*, the probability that the student *j*’ *q*:th occurrence in the segment *k* is $\frac{L_{k}-1}{M-1}$. Thus, the probability of their joint occurrence can be represented as:

$$\begin{array}{c} \;E\left(I_{pqk}\right)=\frac{L_{k}}{M}\frac{L_{k}-1}{M-1}\; \end{array}$$ (2)

Substituting Eqs (2) into (1) gives

$$\begin{array}{c} \;E\left(X_{ij}\right)=\frac{n_{i}n_{j}}{M(M-1)}\sum_{k=1}^{K}L_{k}\left(L_{k}-1\right)\; \end{array}$$ (3)

It is worth mentioning that the expected number of co-occurrences is symmetric with respect to the index of students *i* and *j*, i.e. *E*(*X*_*ij*_) = *E*(*X*_*ji*_). We assume that the number of co-occurrences of two students follow the Poisson distribution very closely when both *n*_*i*_ and *n*_*j*_ are small compared to *K*. A formal proof that this approximation is correct is given provided in the works of Holtsberg et al [24]. We can therefore associate a p-value with the observed number *X*_*ij*_ of co-occurrence as:

$$\begin{array}{c} \;p\left(X_{ij}\right)=\sum_{n=X_{ij}}^{\propto }\frac{E{\left(X_{ij}\right)}^{n}}{n!}e^{-E(X_{ij})}\; \end{array}$$ (4)

To statistically validate the co-occurrence *X*_*ij*_, we use a statistical threshold of 0.01 to check for the statistical significance of the p-values *p*(*X*_*ij*_). However, since the null hypothesis of random co-occurrences is tested for all links of the students’ social network *A*, we need to consider the population parameters while considering the entire set of comparisons. For this, we must perform a multiple hypothesis test correction in order to control the number of false positives. In this work, we use the Bonferroni correction, which is the strictest multiple-hypothesis test correction to minimize the number of false positives [22]. The Bonferroni correction for the multiple testing hypothesis is $p_{b}=\frac{0.01}{N_{T}}$, where *N*_*T*_ is the number of performed tests. For the validation of our student social network, we need to conduct a Bonferroni correction with *N*_*T*_ = 93,529,939. Hence, the Bonferroni correction for the multiple hypothesis test for our dataset is: *p*_*b*_ = 1.07*e*^−10^. If the p-value *p*(*X*_*ij*_) is less than the value of *p*_*b*_ derived above, we conclude that the number of co-occurrences of students *i* and *j* cannot be explained by the null hypothesis that all co-occurrences are due to chance alone. On the contrary, if the test does not reject the null hypothesis, such co-occurrence is due to chance and the link between the students *i* and *j* is removed. Since the resultant student network only contains links which are statistically significant, this network is named the statistically validated co-occurrences network. Our data is a spatio-temporal data stream generated by students, and any spatio-temporal data stream including the basic features generally such as timestamp, location and subject, can also be obtained in the majority of other situation. Furthermore, except using co-occurrences, there is no additional condition in the statistical verification process. Thus, the method proposed here is general, and can be applied easily to any situation with information about the spatio-temporal co-occurrence of interacting agents.

### Method validation

The reliability of existing measures for social ties has been debating in the past few decades [9, 25, 26], In our approach, after successfully deleting the edges generated by chance, whether the SVCN representing true social ties remains unclear. Therefore, the underlying reason for the emergence of these links needs to be confirmed: whether they are true social ties or only same behavior patterns. In order to validate our results, we collected self-report social ties data of 46 students from the College of Science Physical and Technology, where subjects were asked about be familiar with, and friendship with, others. All relevant details on self-report survey data are available in the S1 Text. In our self-report, the acquaintances and friends are referred to as social ties, then we compared the inferred social ties with the real social ties. As depicted in Table 1, the QAP (Quadratic Assignment Procedure) shows that there was a statistically significant and a substantially strong correlation between real social ties and inferred social ties among individuals (*r* = 0.826, *p* < 0.001). If we only consider the friend ties, we found that there was also a strong correlation between real friend ties and inferred social ties(*r* = 0.719, *p* < 0.001).

**Table 1:** The correlations and hit rates between real social ties(friend ties) and inferred social ties.

	*QAP*		*TPR*	*TNR*
	*r*	*p*		
*Real* *social* *ties* & *Inferred* *social* *ties*	0.826	<0.001	0.884	0.960
*Real* *friend* *ties* & *Inferred* *social* *ties*	0.719	<0.001	0.878	0.927

As depicted in Table 1, TPR (True Positive Rate) and TNR (True Negative Rate) measure the hit rate of social ties inferred by our method. In other words, it is possible to accurately infer at least 88.4% of real social ties through our method. For friend ties, the TPR is 0.878, it means that 87.8% of friendships can be inferred. The TNR even reach 0.96, it is possible to accurately infer 96% of individuals who do not have social ties truly. In addition, there were some inferred social ties which did not appear in the real social ties, these students may have the same behavior patterns. Therefore, we believe that the formations of the most links in the SVCN are mainly driven by social ties, while only a very small part of the links are generated due to the same behavior patterns. We call these links generated by social ties or same behavior patterns as social proximity ties.

## Results

### Component sizes and path length

**Fig 1:**
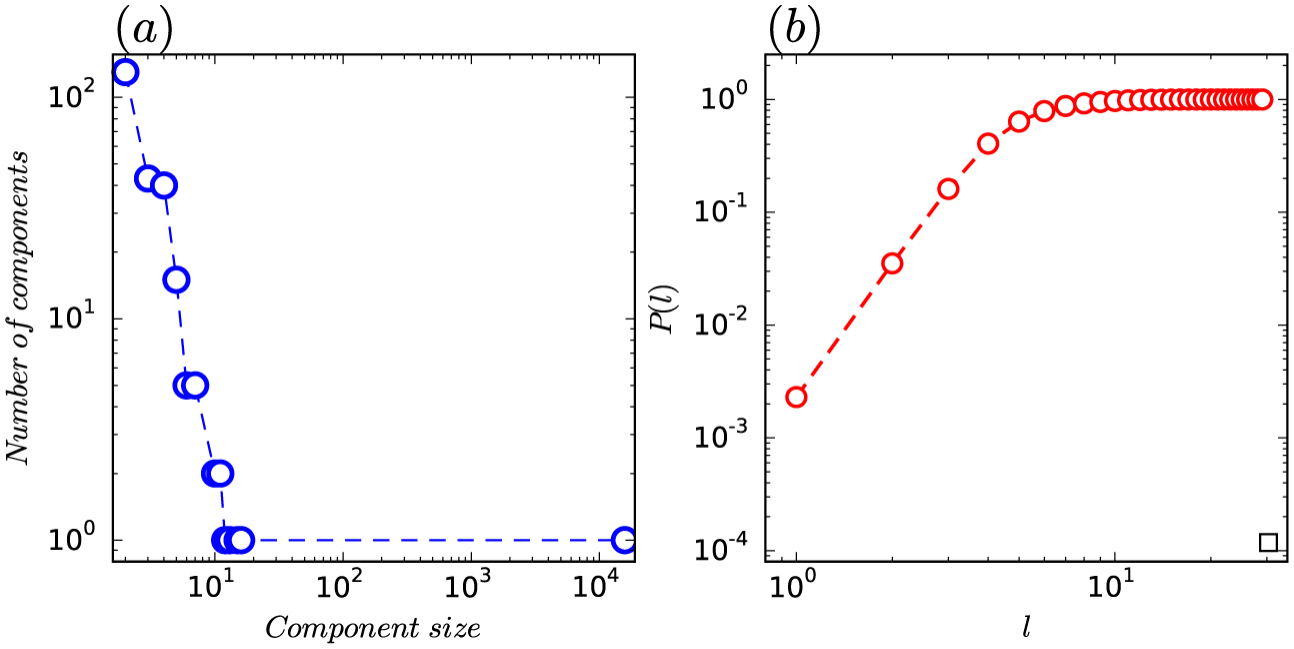
Network components and path length. (a) The fraction of components with a given component size for the SVCN on a log-log scale. (b) The fraction of student pairs *p*(*l*) that are within *l* hops of each other.

Using the method explained in the Materials and Methods section, we obtain a statistically validated co-occurrence network (SVCN) for the academic year from Sept. 2015 to Jun. 2016. This SVCN contains 247 connected components. A connected component is a set of individuals for which each pair of individuals is connected by at least one path through the network. In the SVCN obtained above most of these components are extremely small. Fig 1(a) displays the fraction of components for a given component size on a log-log scale. We can observe that only a few connected components contain a very large number of students and many connected components have very few students. In our SVCN, the largest connected (giant) component has 15,782 students and 278,346 social proximity ties while there are only 16 students and 28 social proximity ties in the second largest connected component. Note that 88.69% of the students and 99.74% of the social proximity ties belong to the giant component. Surprisingly, there are 1,226 singleton students with no social proximity ties in the network, accounting for 6.89% of the total population.

Path length measures the separation between two nodes in the network. We choose to study the size of the neighborhood based on a particular path length, instead of the path length itself. Namely, we use the neighborhood function *NF*(*l*) to describe the number of pairs of nodes (*u*, *v*) such that *u* is reachable from *v* in less that *l* hops. It provides the information about how fast the “average ball” around each node expands. For *l* = 0, we only have the self-pairs: *NF*(0) = *N*, where *N* is the total number of nodes of the SVCN. For the diameter *D* of the SVCN, if *l* = *D*, we have the self-pairs plus all other possible pairs: *NF*(*D*) = *N*^2^, which is the maximum number of possible pairs. We define the fraction of student pairs that are within *l* hops of each other as $p\left(l\right)=\frac{NF(l)}{N^{2}}$. Fig 1(b) plots *p*(*l*) as a function of the hops *l* in log-log scale. It can be seen that *p*(*l*) increases rapidly when *l* ≤ 8 and saturates to 1 with a slower growth rate for *l* > 8. For the SVCN, we also taked a comparison of clustering coefficient and path length to the random null models. The clustering coefficient and the average path length between pairs of students for the SVCN was 0.39 and 5.29. while the random network represents 0.002 and 3.04 respectively. Apparently the SVCN has a much larger clustering coefficient, but when it came to the average path length, we should note that although the SVCN’s was larger(it should be smaller ideally), 5.29 was a proper result when considering the average path length showed a linear increase with the increasing of *log*(*N*) = *log*(17795) ≈ 4.25. Hence, the SVCN exhibits a small-world phenomenon.

### Community structure

**Fig 2:**
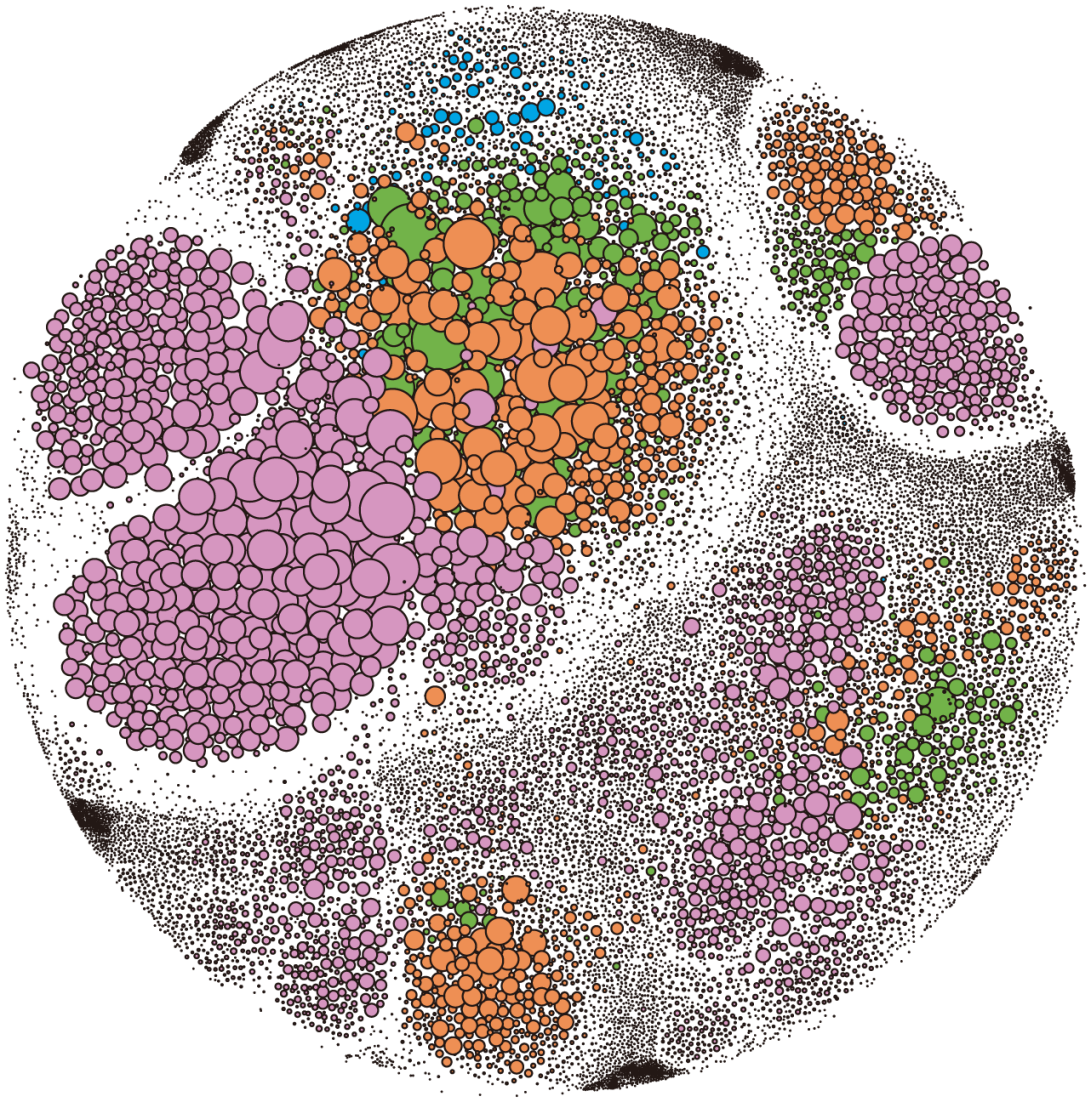
The community structure of the SVCN. To better visualize the SVCN, we used Gephi to draw a force-directed graph based on the ForceAtlas2 algorithm. The color of the node identifies the grade level of the corresponding student, i.e., purple for freshman, orange for sophomore, green for junior, blue for senior respectively.

The application of community detection algorithms (such as OSLOM [27]) to the SVCN, display a clear community structure. In order to better visualize these communities, we use Gephi to draw a force-directed graph (Fig 2) based on the ForceAtlas2 algorithm [28]. Noack [29] has shown that visual proximities indicate the presence of communities. As shown in Fig 2, each node represents a student. The color of the node identifies the grade level of the corresponding student, i.e., purple for freshman, orange for sophomore, green for junior, blue for senior respectively. The size of the node indicates the degree (i.e., the number of social proximity ties). It consists of 443 communities of varying sizes, and the value of modularity (which is an unbiased measure of group proximity as proposed by Newman [30]) is up to 0.706 in our SVCN. This indicates that the SVCN’s community structure’s division into non-overlapping communities is very high. For the all 279066 links, we find that there are 184154 links appearing in the interior of the communities, while the remaining minority links are across different communities. This shows that the SVCN identify many preferential connections inside communities and few preferential connects bridging different communities. In other words, compared with the bridging different communities, students are more likely to form social proximity ties within the communities. Especially for smaller communities, this is more apparent (e.g. For any two communities of which the number of members are both fewer than 10, although there are 300 community pairs connected with each other in the SVCN, there are only 479 links linking them in total.). Moreover, we found that younger students at lower levels tend to form large communities, while students at higher levels mostly form smaller communities. For communities with greater than 100 members, the numbers of occurrences were 19, 14, 14 and 9 for freshmen, sophomores, juniors and seniors respectively. When identifying smaller communities with less than 4 members, the numbers of communities were 3, 32, 43 and 117 for the four groups respectively. This indicates that as students reached higher levels at the university, the larger communities gradually split into smaller ones leading to the formation of several small communities composed of two or three students.

### Degree distribution

In addition to the investigation of the small-world phenomenon and community structure, we also examined the degree distribution of the SVCN. The degree distribution of a network—the probability distribution of the degrees of the nodes (number of connections of a node with other nodes) over the whole network—is an important topological characteristic. It has been shown to have a profound influence on almost every aspect of a network’s structure and function, including clusters, robustness, spreading processes, and many others [31]. We define the degree *d* of a node as the number of its social proximity ties. Let *p*(*d*) be the fraction of individuals with a degree of *d*. The complementary cumulative distribution function (CCDF) is denoted by $P\left(d\right)=\sum_{k=d}^{\infty }p\left(k\right)$, i.e., the fraction of individuals with degree greater than or equal to *d*.

**Fig 3:**
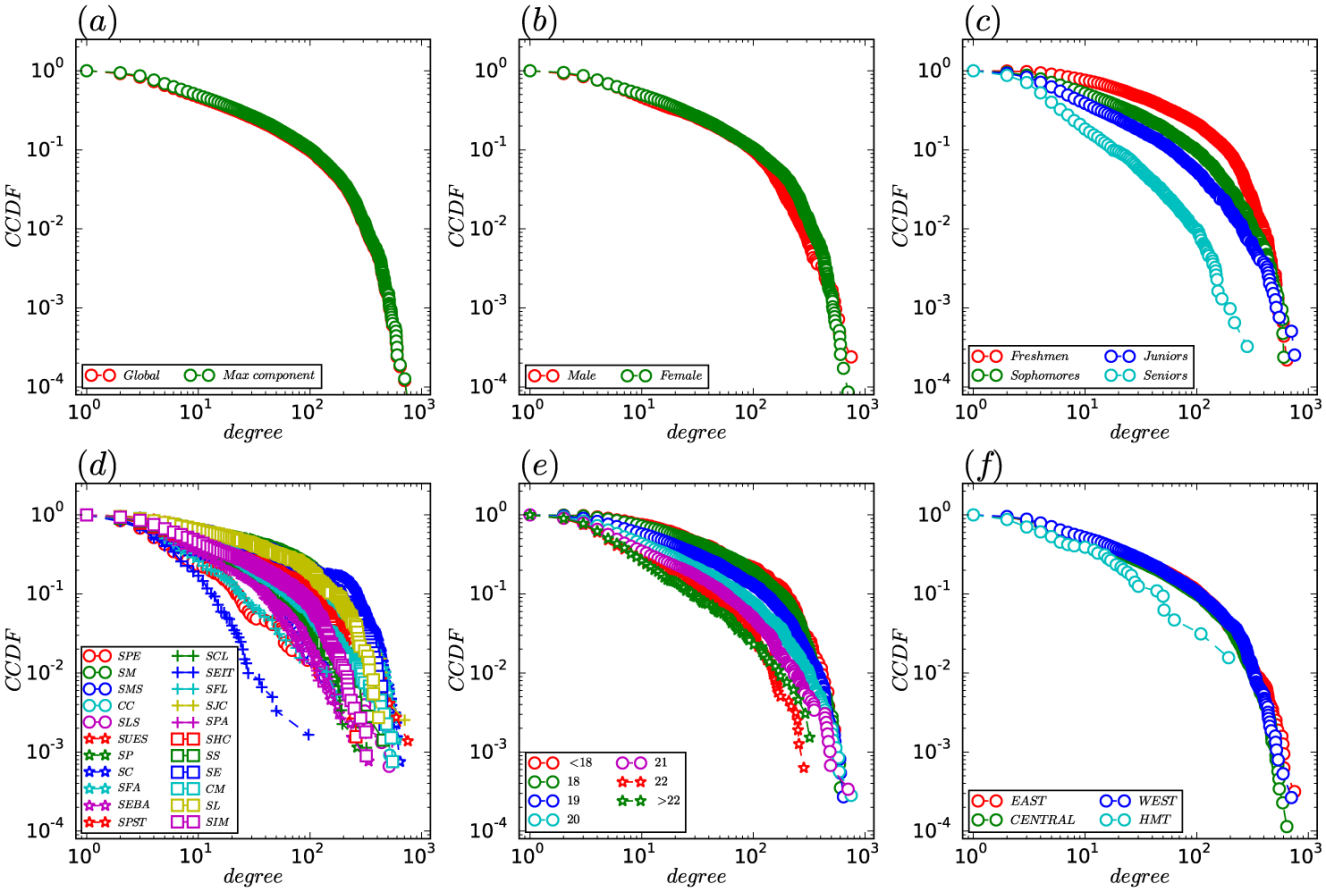
Degree distribution. (a) CCDF of the degree distributions of all students in the SVCN and the giant component. (b) CCDF of the degree distributions for *Male* and *Female*. *Male* denotes the group of male students. The entire list of 2 groups can be found as S1 Table. (c) CCDF of the degree distributions for *Freshmen*, *Sophomores*, *Juniors*, and *Seniors*. *Freshmen* denotes the group of freshman students. The entire list of 4 groups can be found as S2 Table. (d) CCDF of the degree distributions of students from different schools. *SPE* denotes the group of students from the School of Physical Education. The entire list of 22 schools can be found as S3 Table. (e) CCDF of the degree distributions of students of different age groups. *18 years* denotes the group of 18-year-old undergraduate students. The entire list of 7 groups can be found as S4 Table. (f) CCDF of the degree distributions of students from different regions. *EAST* denotes the group of students from eastern region of china. The entire list of 4 groups can be found as S5 Table.

Fig 3(a) plots the CCDF of the degree distributions of the SVCN and that of the giant component of the SVCN. Since the distribution of the giant component is quite similar to that of the SVCN, we focus our attention on the giant component. We observe that the distribution is not straight on a log-log plot and decays faster than a power law distribution. We use the maximum likelihood fitting method [32] to fit the distribution -the result of which suggests that the distribution is best fitted by an exponentially truncated power law $P(d)\propto d^{-\infty }exp\left(\frac{-d}{d_{c}}\right)$ with a power-law exponent *α* = 1.05 and a cutoff degree *d*_*c*_ = 159.74. The power-law exponent *α* reflects the extent that the degrees of the nodes span within a network. The cutoff degree *d*_*c*_ is a critical value. If *d* < *d*_*c*_, it is almost identical to a normal power-law, and if *d* > *d*_*c*_, we get a normal exponential decay. In our SVCN, we observe a strong cutoff degree and low power-law exponent, suggesting a distribution with high variance.

Fig 3(b) shows the CCDF of the degree distributions for *Male* and *Female*. The distributions have curved shapes on a log-log plot, indicating an accelerated decay for higher values of *d*. These distributions also follow the exponentially truncated power law. The estimated parameters are *α* = 1.00 and *d*_*c*_ = 143.06 for *Male*, *α* = 1.00 and *d*_*c*_ = 165.84 for *Female*. The degree distributions for *Male* and *Female* show minor differences. Moreover, the average degree of *Male* is close to that of *Female*. From this we can conclude that there is only a minor difference in the number of social proximity ties among the genders.

In Fig 3(c), we present the CCDF of the degree distributions for *Freshmen*, *Sophomores*, *Juniors*, and *Seniors*. These degree distributions can also be well fitted by the exponentially truncated power law. The estimated parameters are: *α* = 1.00 and *d*_*c*_ = 336.70 for *Freshmen*, *α* = 1.00 and *d*_*c*_ = 157.73 for *Sophomores*, *α* = 1.08 and *d*_*c*_ = 116.01 for *Juniors*, *α* = 1.28 and *d*_*c*_ = 429.16 for *Seniors*. It is remarkable that there are clear differences in the values of *α* and *d*_*c*_ between the different grades. Additionally, the average degrees of the four groups are 60.12, 34.92, 23.35 and 8.7 respectively. One explanation for this observation could be that the social proximity ties gradually stabilize over time and some fragile social proximity ties disappear in higher grades.

Fig 3(d) displays the CCDF of the degree distributions of students from different schools. While these degree distributions exhibit the same, truncated power-law, behavior with values of exponent *α* between 1.00 and 1.35, there are obvious observable differences in the degree distributions of students from different schools. Take *SEIT* as an example—its curve of degree distribution (shown by the blue ‘+’ symbol in Fig 3(d)) is significantly different from that of the other schools. Its power-law exponent *α* is the largest with a value of 1.35, which leads to a steeper slope. The estimated cutoff degree of *SEIT*, *d*_*c*_, is significantly smaller than that of other schools. This is also evident from its curve where for a degree *d* > 14, there is a significant drop in the value of CCDF. That is, the combined effect of these two parameters leads to a narrower degree distribution than that of other schools. This indicates that the students from *SEIT* have a lower average number of social proximity ties than the students from other schools.

In Fig 3(e), we show the CCDF of the degree distributions of students of different age groups: *<18 years*, *18 years*, *19 years*, *20 years*, *21 years*, *22 years* and *>22 years*. These distributions can also be nicely fitted by the exponentially truncated power law. The power-law exponent *α* stabilizes at about 1.00 until *19 years*, and gradually increases after *19 years*. The cutoff degree *d*_*c*_ significantly reduces from 340.13 in *<18 years* to 29.67 in *>22 years*. The average degree also significantly reduces from 63.52 in *<18 years* to 13.43 in *>22 years*. We can observe that the older the students, the fewer social proximity ties they had.

Fig 3(f) displays the CCDF of the degree distributions of students from different regions. According to the geographic location and the level of economic development, mainland China was classified into three regions. We also consider Hong Kong, Macao and Taiwan (*HMT*). The degree distributions of *EAST*, *CENTRAL*, and *WEST* display a certain similarity, although the tails for *EAST* and *WEST* are longer than that for *CENTRAL*. The degree distribution of *HMT* has a steeper slope and therefore has a narrower distribution of degrees than that of other regions. It may be that some students who are short-term exchange students have not adapted to life in the mainland China very well, and hence they have fewer social proximity ties.

### Degree assortativity

Many social networks show “assortative mixing” on their degrees, i.e., a preference of high-degree nodes to connect to other high-degree nodes. In order to quantify the global connectivity tendency of the SVCN, we use the *global degree assortativity coefficient* [33] *r* which is the Pearson correlation coefficient of the degrees of the pairs of nodes of all the edges in the network. An assortativity coefficient of greater than zero implies that large-degree nodes are typically connected with each other, and the network is assortative. On the other hand, a value less than zero indicates that the network is disassortative. In our SVCN, the value of *r* ≈ 0.42, implying that there are a high number of connections between the nodes with high degrees. In other words, the students with many social proximity ties connect with each other to form the backbone of the small-world network. This enables the highly clustered students at the edge of the SVCN to achieve low average path lengths to the students in the rest of the network.

**Fig 4:**
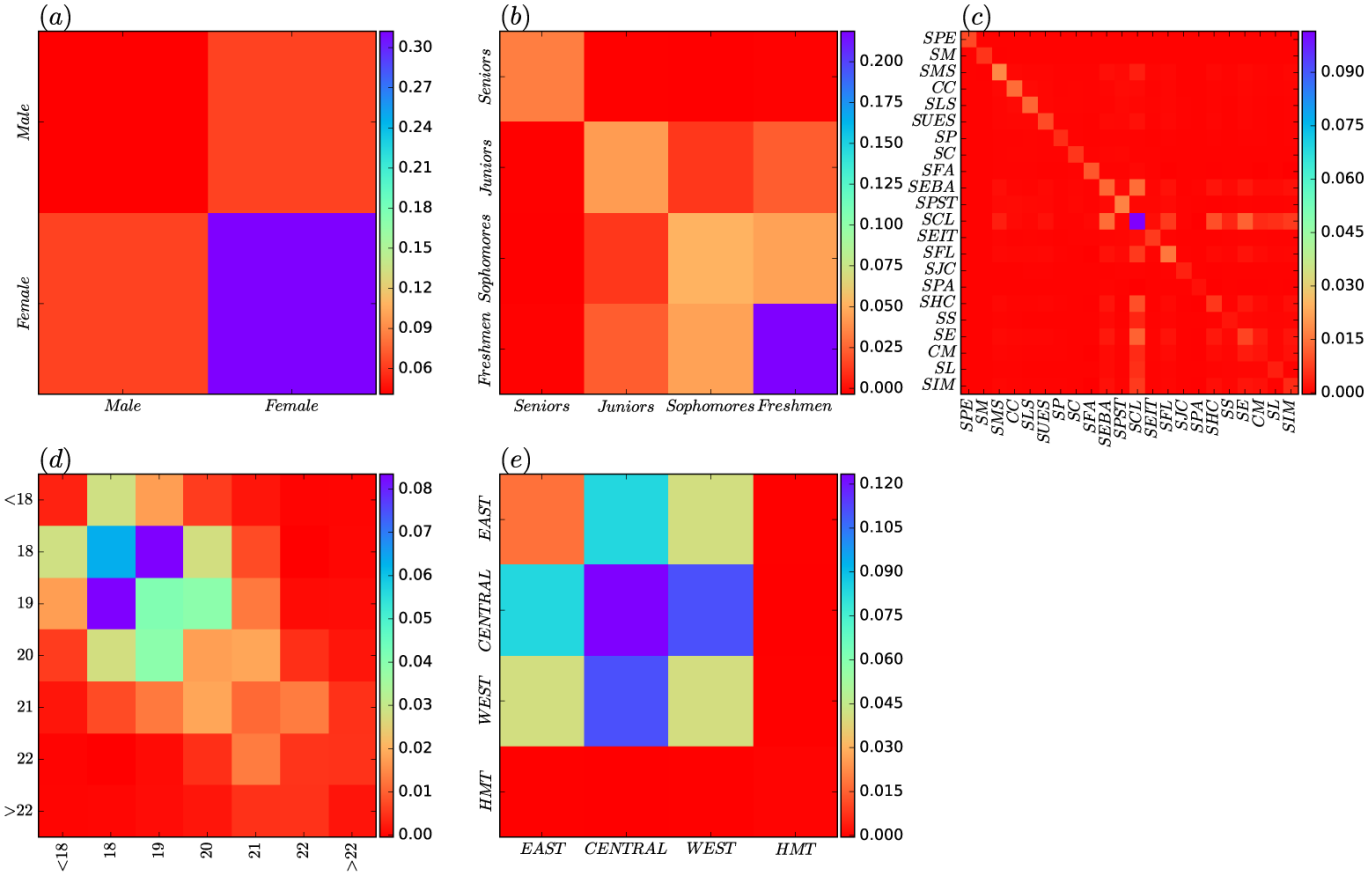
Degree assortativity. (a) the intra- and inter-gender degree assortativity coefficient. (b) the intra- and inter-grade degree assortativity coefficient. (c) the intra- and inter-school degree assortativity coefficient. (d) the intra- and inter-age degree assortativity coefficient. (e) the intra- and inter-region degree assortativity coefficient.

To further investigate the degree assortativity of the SVCN from a mesoscale level, we use the *universal assortativity coefficient* [34] to measure the intra- or inter-group connection tendencies. Here, these groups are used to partition the SVCN according to student attributes such as gender, grade, school, age and region. Fig 4(a) shows the universal assortativity coefficient matrices for gender. In this figure, different colors are used to discretize the strength of assortativity within and between genders. The matrices provide two comparisons within gender: *r*(*Male*, *Male*) and *r*(*Female*, *Female*). We can observe that the value of *r*(*Female*, *Female*) is higher than that of *r*(*Male*, *Male*), which means that the number of social proximity ties between popular female students (female students with high degrees) tend to be more than those between popular male students. For across genders connections, the matrices are symmetric so that the *r*(*Male*, *Female*) equals *r*(*Female*, *Male*). We found that the assortativity across genders is stronger than that of male-to-male, but weaker than that of female-to-female.

Fig 4(b) shows the universal assortativity coefficient matrices of the different grades. Compared with the students of other grades, the intra-grade assortativity coefficient of *Freshmen* was significantly higher (even > 0.20), implying that popular freshmen connect with each other very frequently. Next, we observe that the intra-grade assortativity coefficient decreases as the grade level increases. This indicates the tendency of popular students to establish social tie with other popular students decreases as they go from a low grade to a high grade. For inter-grades connections, a comparison of inter-grade assortativity shows the following pattern: *r*(*Juniors*, *Freshmen*) > *r*(*Juniors*, *Sophomores*) > *r*(*Juniors*, *Seniors*), which indicates that large-degree juniors tend to associate more with large-degree freshmen. *Sophomores* also exhibit a similar phenomenon with *Juniors*.

As shown in Fig 4(c), the intra- and inter-school degree assortativity coefficients are small, the maximum of which is only 0.09 for SCL. Therefore, we believe that the intra- and iner-school connections do not show any patterns of assortativity. The Similar phenomenon can also be found in Fig 4(d), which infers that the number of social proximity ties has little privilege for the different age groups of students to make friends. Only the 18 years and 19 years have very little value of inter-age assortativity and intra-age assortativity.

The Fig 4(e) displays the intra- and inter-region degree assortativity. Within the region, the assortativity of *CENTRAL* is most obvious, the *r*(*CENTRAL*, *CENTRAL*) is almost 0.12. From the horizontal column, we can observe that the assortativity coefficients between *CENTRAL* and other regions are also significantly higher than those between the other regions—the value of *r*(*CENTRAL*, *EAST*) is greater than 0.07 and that of *r*(*CENTRAL*, *WEST*) is greater than 0.10. This shows that students from the central region of China are more likely to socialize with similar students regardless of their region. The *EAST* and *WEST* showed a similar degree assortativity pattern with *CENTRAL*, but a lower assortativity coefficient than *CENTRAL*. For *HMT*, it does not show assortativity within or between regions.

### Attribute assortativity

From a more general point of view, assortativity indicates the psychological tendency that people prefer to form ties with others having the same or similar attributes. Those attributes can either be categorical (such as school, gender and grade) or scalar (e.g., age). In social studies, assortativity refers to homophily [35]. Empirical evidence exists to show that individuals demonstrate significant homophilic tendencies in different social networks, ranging from friendship networks to business relationships networks [33, 36–39].

We first use the *attribute assortativity coefficient* [33, 35] to measure the global connectivity tendency of the SVCN which is divided into different groups respectively according to gender, grade, school, age and region. For each tie *l*, its two endpoints have values of *x*_*l*_ and $x_{l}^{{}^{\prime }}$ respectively for the scalar attribute *x*. The Newman’s assortative coefficient is given by:

$$\begin{array}{c} \;r_{x}=\frac{\sum x_{l}x_{l}^{{}^{\prime }}\left[p\left(x_{l},x_{l}^{{}^{\prime }}\right)-p\left(x_{l}\right)p\left(x_{l}^{{}^{\prime }}\right)\right]}{\sigma_{x}\sigma_{x^{{}^{\prime }}}}\; \end{array}$$ (5)

where *σ*_*x*_ is the variance of *x*; $p(x_{l},x_{l}^{{}^{\prime }})$ is the probability that there is a tie between two individuals with the attribute values of *x*_*l*_ and $x_{l}^{{}^{\prime }}$. The value is *r*_*x*_ = 1 when there is a perfect assortative mixing, i.e., all individuals associate solely with others of the same type as themselves. *r*_*x*_ = −1 indicates perfect disassortative mixing, which is the exact opposite of the previous situation; *r*_*x*_ = 0 indicates that the mixing is completely random. A similar expression exists for the case where an individual *i* is associated with a categorical attribute.

We calculate the attribute assortativity coefficient based on the gender, grade, school, age, and region of the students. The SVCN is highly assortative by grade and school with the assortativity coefficients *r*_*grade*_ = 0.64 and *r*_*school*_ = 0.58. It implies that the majority of students have formed social proximity ties with other students of the same grade or school. In other words, the grade or school has a very big influence on the overall topology of the SVCN, compared with the other attributes. It may be due to the reason that students enrolled in the same year or in the same school usually attend in the same class, or live together, which provides opportunities for face-to-face interactions among these students. Assortative mixing based on age and gender is common in human social networks. We also find that the SVCN is assortative by age and gender (e.g. *r*_*age*_ = 0.45, *r*_*gender*_ = 0.39), although the mixing is not as strong as that by grade or school. A small assortativity coefficient for region (*r*_*region*_ = 0.05) shows that the SVCN is less assortative by region.

To further investigate the preference for the intra- and inter-group tie formation, we consider tie patterns amongst students of different attributes. Here, we want to consider the SVCN for a certain attribute. Let *N* be the total number of students in the SVCN. In order to measure the intra- and inter-group connectivity tendencies, the SVCN is divided into *k* groups according to this attribute. The number of students in group *i* is denoted by *N*_*i*_, where *i* = 1, …, *K*. Hence, the total number of students in all groups is given by *N* = ∑_*i*_ *N*_*i*_. The fraction of a population that present in group *i* is denoted by $w_{i}=\frac{N_{i}}{N}$. Let *s*_*ij*_ denote the average number of links between the students in group *i* and the students in group *j*. The average number of links that a student in group *i* has is $s_{i}=\sum_{j=1}^{k}s_{ij}$. Referring to the homophily index [40], the average fraction of the member of group *j* in all the links of group *i* is represented as:

$$\begin{array}{c} \;p_{ij}=\frac{s_{ij}}{s_{i}}\; \end{array}$$ (6)

However, *p*_*ij*_ does not provide us the measure of the inter- and intra-group connectivity tendency and can’t fully capture how potentially biased a group could be in a network. Hence, similar to the inbreeding homophily index [35], to take care of this and normalize the homophily index we define the *tie formation preference index* (TFPI) as:

$$\begin{array}{c} \;q_{ij}=\frac{p_{ij}-w_{j}}{1-w_{j}}\; \end{array}$$ (7)

Note that, if *P*_*ij*_ = *w*_*i*_, then there is no connection preference between group *i* and group *j*, then, *q*_*ij*_ = 0. If group *i* shows more preferences than the random to connect with group *j*, there is *q*_*ij*_ > 0. Conversely, *q*_*ij*_ < 0. Note that when *i* ≠ *j*, the *q*_*ij*_ stands for inter-group TFPI, when *i* = *j*, it represents intra-group TFPI.

**Fig 5:**
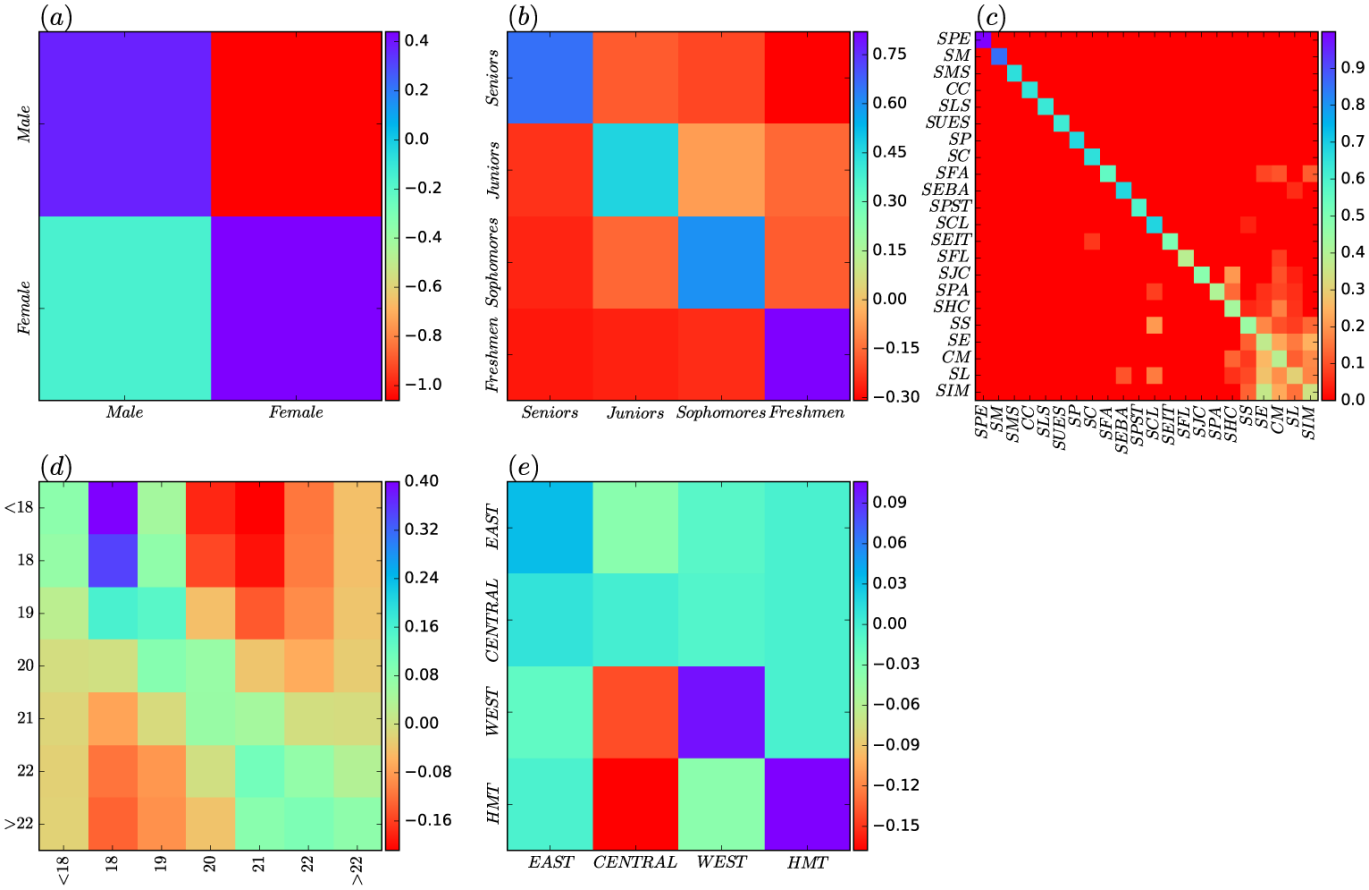
Attribute assortativity. (a) the intra- and inter-gender ties formation preference indices (TFPI). (b) the intra- and inter-grade TFPI. (c) the intra- and inter-school TFPI. (d) the intra- and inter-age TFPI. (e) the intra- and inter-region TFPI.

Fig 5(a) shows the intra- and inter-gender *tie formation preference index* (TFPI). Several clear patterns can be observed from the plot. Firstly, the connection preferences within gender are apparent (cf. the values of TFPIs for *Male-Male* and *Female-Female* are both positive), which means that intra-gender social proximity ties are more to form than expected. Secondly, the value of TFPI for *Female-Female* is greater than that for *Male-Male*. This indicates that, compared to males, females are more likely to form internal social proximity ties each other. Thirdly, we find that the values of TFPIs for *Male-Female* and *Female-Male* are both negative. This means that there are a few ties across the genders than expected. Finally, the value of TFPI for *Male-Female* is smaller than that for *Female-Male*, which means the connections from males to females is lower than that from females to males. In other words, this may imply that males are more reluctant to establish social proximity ties across genders.

Switching to grade, the Fig 5(b) shows the intra- and inter-grade TFPI values. We show that all grades demonstrate strong intra-grade connection preferences. Specially, the connection preference within *Freshmen* is the strongest, with the value of TFPI exceeding 0.75. Then, the values decrease monotonically as the grade increase. This may reveal a phenomenon: As the freshmen enter the new campus environment, they are still in the adaptation period, with the formation of social proximity ties among them being confined to the within their grade. As they gradually adapt to the environment, their connection preferences change, and this change is further evident as they become juniors. However, the value of intra-grade TFPI for *Seniors* increases significantly. A possible cause for this is that the seniors have formed relatively stable intra-grade social proximity ties over time as they often engage in some common activities together.

Fig 5(c) reports the intra- and inter-school TFPIs for 22 schools. All schools show strong intra-school connection preferences, as illustrated by the anti-diagonal in the plot. Specially, the values of TFPIs even reach 0.90 or more for *SPE* and *SM*, indicating that the students in the two schools form social proximity ties almost only within school. For *SPE*, this may be due to the fact that students are on a separate campus, so they have less access to contact with students from other schools. For *SM*, this could be because of the professional nature of the school—the students have a lot of professional performance activities, and fewer inter-school social activities. Moreover, as illustrated by an eye-catching square area at the bottom right corner of Fig 5(c), for a few schools, namely, *SS*, *SE*, *CM*, *SL* and *SIM*, the values of their inter-school TFPIs are greater than zero, so there are a large number of inter-school connections. Note these schools belong to humanities, social-sciences or similar categories. This may imply that students in these schools not only are inclined to establish intra-school social proximity ties, but also are willing to form social proximity ties among peers of similar categories.

From Fig 5(d), we can observe that the age-based (*<18 years*, *18 years*, *19 years*, *20 years*, *21 years*, *22 years* and *>22 years*) connection preferences are apparent since the values of the TFPIs are all positive. This shows that students generally establish social proximity ties with the students of similar ages. Moreover, as illustrated by the one eye-catching purple grid in the upper left corner of this plot, the inter-age connection preference from the *<18 years* to *18 years* is very obvious and the value of TFPI peaks at about 0.40. This means that students under 18-year-old have significant preferences to form social proximity ties with students aged 18.

Fig 5(e) displays the intra- and inter-region connection preferences. In addition to *CENTRAL* and *EAST* with very weak intra-region connection preferences (the values of TFPIs close to 0.09), the values of intra- and inter-region TFPIs for all regions are all close to zero. Because of the regional division based on geographical location, we can hypothesize that their choice of social proximity ties is independent of the region of the other students. As illustrated by the two red grids in this plot, students from central region are less popular with students from western region and Hong Kong, Macao and Taiwan.

## Discussion

In this paper, we studied how to find the underlying social network structure of a population that can only be observed through the spatial trajectories of its individual members. We used as a case study a setting where the students scan their student cards at various sites on the campus and their spatial trajectories are left behind in the form of a long time-stamped check-in data stream. By assuming that social tie is a latent factor influencing whether or not the two students access the time-stamped location together (i.e., co-occurrences), we proposed a methodology, based on statistical validation, to extract social proximity ties from those spatio-temporal data stream. In our approach, links were drawn between students based on their co-occurrences. By assuming that the number of co-occurrences follows the Poisson distribution, we validated each link against the null hypothesis that all co-occurrences are due to chance alone.

We then studied the structure of the social network of students using many metrics and tools. We characterized the global structure of the network. The small-world phenomenon was then confirmed on a truly global scale. We interpreted this result as indicating that any student only need to advance a few steps across the SVCN to establish some indirect social proximity ties with a substantial fraction of the all students. We found that the network showed clear community structure. And we observed that younger students at lower levels tended to form large communities, while students at higher levels mostly formed smaller communities. Apparently, the distribution of the number of the students’ social proximity ties varied significantly on different attributions, and it was quite easier to form social proximity ties intra-grade and intra-school.

Switching to assortativity patterns, we observed clear degree assortativity on a global scale. We also discovered that degree assortativity had some idiosyncratic characteristics on a local scale. Taking gender as example, we observed that the number of social proximity ties between popular female students tended to be more than those between popular male students. Furthermore, we found that the assortativity across genders was stronger than that of male-to-male, but weaker than that of female-to-female. For attribute assortativity, we observed a strong effect of grade and school on tie formation preferences, but we did not find any strong region homophily.

This study has several implications. Firstly, we constructed social network from spatio-temporal data including discretizing the observation stream based on a fixed time window. An inappropriately small time window may lead to the SVCN that could not capture important links, while a very large time window would overload the SVCN with noise links. Future research should address these limitations by using the clustering scheme that allows us to automatically infer the appropriate length of time windows [41]. Secondly, due to the data is scattered across departments, we only collected the check-in records of students in the canteens and stores. It may only reveal relatively single social ties − we may miss social ties between students who meet at other places such as libraries. In the future research, we will collect the check-in records from multiple departments and reveal multi-dimensional social ties. Finally, social ties are usually not static structures, meaning that people maintain a series of types of ties and these ties evolve with time. Therefore, we will expand our research to study the evolutionary nature of social networks.

## Supporting information

S1 Text Additional details on the data. In this Supporting File we provide all relevant details on data collection, including an explanation of the data collection process, data format description, data availability, data grouping, and self-report survey data.
(PDF)

S1 Fig The RFID tags deployment diagram. The RFID tags are armed in 10 canteens and 7 stores all around the school, we extracted 9,147,106 pieces of data from 17,795 students containing the freshman, sophomore, junior and senior during one academic year (from September, 2015 to June, 2016). When students go to the student canteens for dinner, they should have their card scanned to pay for the meal.
(TIF)

S1 Table Groups by gender. According to the gender attribute of students, the node of the SVCN is divided into two groups: *Male* and *Female*. Followed by the fraction of *Male* and *Female* in the SVCN respectively.
(PDF)

S2 Table Groups by grade. According to the grade attribute of students, the node of the SVCN is divided into four groups: *Freshmen*, *Sophomores*, *Juniors* and *Seniors*. Followed by the fraction of *Freshmen*, *Sophomores*, *Juniors* and *Seniors* in the SVCN respectively.
(PDF)

S3 Table Groups by school. According to the school attribute of students, the node of the SVCN is divided into twenty two groups. Followed by the fraction of twenty two groups in the SVCN respectively.
(PDF)

S4 Table Groups by age. According to the age attribute of students, the node of the SVCN is divided into seven groups. Followed by the fraction of seven groups in the SVCN respectively.
(PDF)

S5 Table Groups by region. According to the region attribute of students, the node of the SVCN is divided into three groups. We also consider Hong Kong, Macao and Taiwan, referred to as *HMT*. Finally,the fraction of *EAST*, *CENTRAL*, *WEST* and *HMT* in the SVCN respectively.
(PDF)
