# Supplementary material for: Inferring and analysis of social networks using RFID check-in data in China
Source: PLoS One. 2017 Jun 1;12(6):e0178492. doi: 10.1371/journal.pone.0178492 (PMC5453530; doi:10.1371/journal.pone.0178492)
Supplement: S1 Text — In this Supporting File we provide all relevant details on data collection, including an explanation of the data collection process, data format description, data availability, data grouping, and self-report survey data. (PDF) [file pone.0178492.s001.pdf]

Supporting Information

S1 Text

Below we provide all relevant details on data collection, including an explanation of the data collection process, data format description, data availability, data grouping, and self-report survey data.

Data Acquisition. The check-in data was collected from the Student Card System (SCS), at the Central China Normal University, Hubei, China. Student Card System (SCS) is one share of the construction of smart campus in China. The system includes three components, which are RFID tags system, student card and the database. Once a student enrolled, he/ she would be assigned a unique student number, which serves as the ID around the campus. Each student will also have a unique card barcoded with student number. This so called "Student Card" is a magnetic stripes card which functions as ID card, stored-value card and consumer card on campus. RFID tags are armed in several locations such as student canteens, dormitories, library, classrooms and stores across the university campuses. Generally, almost every campus activity obtains a student card check-in. For instance, a student wants to enter the library, his card should be scanned, thus this library check-in data, which includes the information of student number, location (library) and time record, would upload to the database. In this way, the university database has recorded most of the student campus activities.

The work described here is a part of data we extracted from SCS database, including activities occurred in canteens and stores. When students go to the student canteens for dinner, they should have their card scanned to pay for the meal. Since the RFID tags are armed in 10 canteens and 7 stores all around the university campuses (as is shown in S1 Fig), we extracted 9,147,106 pieces of data from 17,795 students containing the freshman, sophomore, junior and senior during one academic year (from September, 2015 to June, 2016). Each entry of the records contains the following information: *student ID*, *location* (canteen's or store's number) and *timestamp* (the time that the activity occurred).

Data Format. Full details on the data format of the students' consumption records in canteens and department stores.

$(STUDENTID)$ 10000 $(LOCATION)$ 12 $(TIMESTAMP)$ $2015-10-12\ 12:30:22$

Data availability. The authors confirm that all data underlying the findings are fully available without restriction. All the anonymized version of this dataset can be found via Figshare ($http://dx.doi.org/10.6084/m9.figshare.4880000$ and $http://dx.doi.org/10.6084/m9.figshare.4879985$).

Data Grouping. According to the attribute (e.g. gender, grade, department, age and region) of students, the node of the SVCN is divided into different groups, followed by the abbreviation of each group and the fraction of the number of students in each group for the SVCN respectively. Details see in the S1-S5 Tables.

Self-Report Survey Data. At the end of the one year study we conducted a self-report survey for 46 of the all subjects according to the different inference results. This survey included three questions regarding the real friendship with the other subjects. The questions used for this analysis are written below.

The ID and name of subjects: 10000 *Liu Da*

Please answer the following questions according to the actual situation, and fill your answer in the right bracket!!

A : 10001 *Zhang San*

B : 10002 *Li Si*

C : 10003 *Wang Wu*

D : 10004 *Zhao Liu*

◊*For the students listed above, do you know them? Please fill the brackets with their student ID.* [ ]

◊*Among these students, which are your friends? According to the degree of intimacy from strong to weak, please fill the brackets with your friends' student ID.* [ ]

◊*If some of your friends are not listed above, please fill in the name and his/her student ID on the following line.*
