# Supplementary figures and images for: Inferring and analysis of social networks using RFID check-in data in China
Source: PLoS One. 2017 Jun 1;12(6):e0178492. doi: 10.1371/journal.pone.0178492 (PMC5453530; doi:10.1371/journal.pone.0178492)

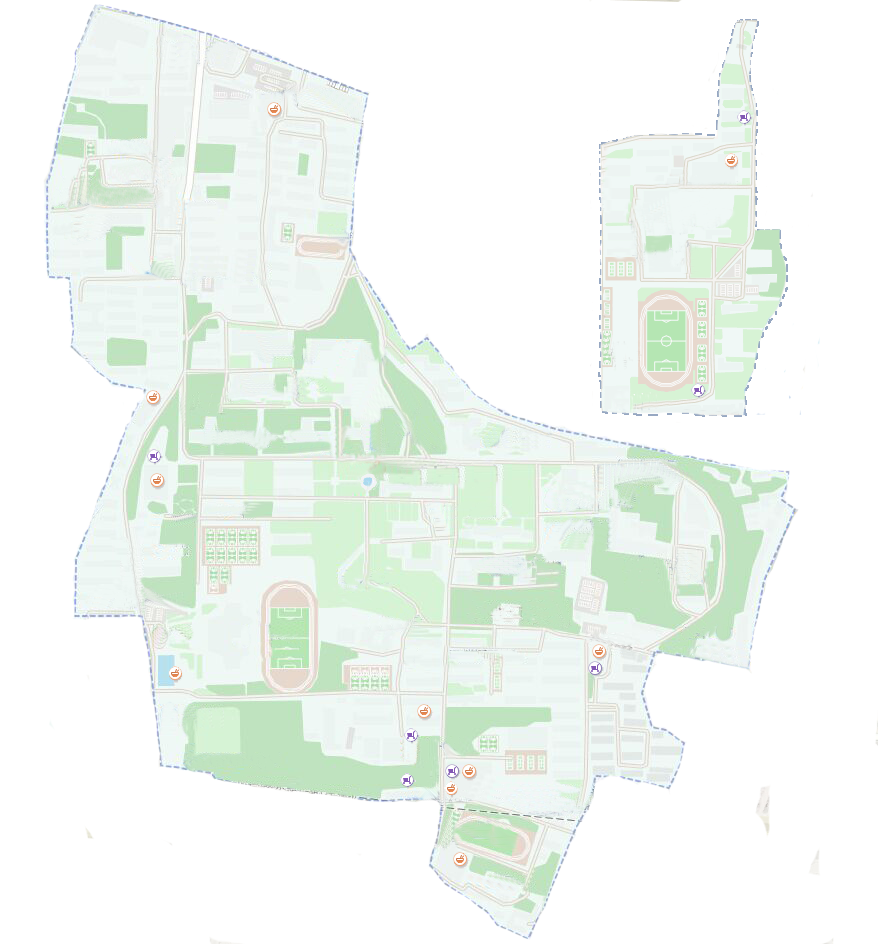

Supplement: S1 Fig — The RFID tags are armed in 10 canteens and 7 stores all around the school, we extracted 9,147,106 pieces of data from 17,795 students containing the freshman, sophomore, junior and senior during one academic year (from September, 2015 to June, 2016). When students go to the student canteens for dinner, they should have their card scanned to pay for the meal. (TIF) [file pone.0178492.s002.tif]
