# Supplementary material for: Inferring and analysis of social networks using RFID check-in data in China
Source: PLoS One. 2017 Jun 1;12(6):e0178492. doi: 10.1371/journal.pone.0178492 (PMC5453530; doi:10.1371/journal.pone.0178492)
Supplement: S1 Table — According to the gender attribute of students, the node of the SVCN is divided into two groups: Male and Female. Followed by the fraction of Male and Female in the SVCN respectively. (PDF) [file pone.0178492.s003.pdf]

full name of gender group	abbreviation	fraction
the male students group	*Male*	0.269
the female students group	*Female*	0.731

S1 Table. Groups by gender. According to the gender attribute of students, the node of the SVCN is divided into two groups: *Male* and *Female*. *Male* denotes the group of male students, and *Female* denotes the group of female students. Followed by the fraction of *Male* and *Female* in the SVCN respectively
