# Supplementary material for: Inferring and analysis of social networks using RFID check-in data in China
Source: PLoS One. 2017 Jun 1;12(6):e0178492. doi: 10.1371/journal.pone.0178492 (PMC5453530; doi:10.1371/journal.pone.0178492)
Supplement: S2 Table — According to the grade attribute of students, the node of the SVCN is divided into four groups: Freshmen, Sophomores, Juniors and Seniors. Followed by the fraction of Freshmen, Sophomores, Juniors and Seniors in the SVCN respectively. (PDF) [file pone.0178492.s004.pdf]

| full name of grade group | abbreviation | fraction |
|---|---|---|
| the freshman group | *Freshmen* | 0.259 |
| the sophomore group | *Sophomores* | 0.249 |
| the junior group | *Juniors* | 0.252 |
| the senior group | *Seniors* | 0.241 |

**S2 Table. Groups by grade.** According to the grade attribute of students, the node of the SVCN is divided into four groups: *Freshmen*, *Sophomores*,*Juniors* and *Seniors*. *Freshmen* denotes the group of freshman students, *Sophomores* denotes the group of sophomore students, *Juniors* denotes the group of junior students and *Seniors* denotes the group of senior students. Followed by the fraction of *Freshmen*, *Sophomores*, *Juniors* and *Seniors* in the SVCN respectively
