# Supplementary material for: Inferring and analysis of social networks using RFID check-in data in China
Source: PLoS One. 2017 Jun 1;12(6):e0178492. doi: 10.1371/journal.pone.0178492 (PMC5453530; doi:10.1371/journal.pone.0178492)
Supplement: S3 Table — According to the school attribute of students, the node of the SVCN is divided into twenty two groups. Followed by the fraction of twenty two groups in the SVCN respectively. (PDF) [file pone.0178492.s005.pdf]

full name of school group	abbreviation	fraction
the students from School of Physical Education	SPE	0.056
the students from School of Music	SM	0.040
the students from School of Mathematics and Statistics	SMS	0.089
the students from College of Chemistry	CC	0.065
the students from School of Life Sciences	SLS	0.052
the students from School of Urban and Environmental Sciences	$SUES$	0.044
the students from School of Psychology	SP	0.021
the students from School of Computer	SC	0.038
the students from School of Fine Arts	SFA	0.042
the students from School of Economics and Business Administration	$SEBA$	0.067
the students from College of Physical Science and Technology	$CPST$	0.078
the students from School of Chinese Language	SCL	0.082
the students from School of Educational Information Technology	$SEIT$	0.027
the students from School of Foreign Languages	SFL	0.079
the students from School of Journalism and Communication	SJC	0.028
the students from School of Public Administration	SPA	0.011
the students from School of Sociology	SS	0.023
the students from School of Education	SE	0.043
the students from School of History and Culture	SHC	0.043
the students from College of Marxism	CM	0.026
the students from School of Law	SL	0.022
the students from School of Information Management	SIM	0.023

S3 Table. Groups by department. According to the school attribute of students, the node of the SVCN is divided into twenty two groups: SPE, SM, SMS, CC, SLS, $SUES$, SP, SC, SFA, $SEBA$, $CPST$, SCL, $SEIT$, SFL, SJC, SPA, SS, SE, SHC, CM, SL and SIM. SPE denotes the group of students from School of Physical Education, SM denotes the group of students from School of Music, SMS denotes the group of students from School of Mathematics and Statistics, CC denotes the group of students from College of Chemistry, SLS denotes the group of students from School of Life Sciences, $SUES$ denotes the group of students from School of Urban and Environmental Sciences, SP denotes the group of students from School of Psychology, SC denotes the group of students from School of Computer, SFA denotes the group of students from School of Fine Arts, $SEBA$ denotes the group of students from School of Economics and Business Administration, $CPST$ denotes the group of students from College of Physical Science and Technology, SCL denotes the group of students from School of Chinese Language, $SEIT$ denotes the group of students from School of Educational Information Technology, SFL denotes the group of students from School of Foreign Languages, SJC denotes the group of students from School of Journalism and Communication, SPA denotes the group of students from School of Public Administration, SS denotes the group of students from School of Sociology, SE denotes the group of students from School of Education, SHC denotes the group of students from School of History and Culture, CM denotes the group of students from College of Marxism, SL denotes the group of students from School of Law and SIM denotes the group of students from School of Information Management. Followed by the fraction of twenty two groups in the SVCN respectively
