# Supplementary material for: Inferring and analysis of social networks using RFID check-in data in China
Source: PLoS One. 2017 Jun 1;12(6):e0178492. doi: 10.1371/journal.pone.0178492 (PMC5453530; doi:10.1371/journal.pone.0178492)
Supplement: S4 Table — According to the age attribute of students, the node of the SVCN is divided into seven groups. Followed by the fraction of seven groups in the SVCN respectively. (PDF) [file pone.0178492.s006.pdf]

full name of age group	abbreviation	fraction
the group of Under 18-year-old undergraduate students	< 18 *years*	0.030
the group of 18-year-old undergraduate students	18 *years*	0.162
the group of 19-year-old undergraduate students	19 *years*	0.215
the group of 20-year-old undergraduate students	20 *years*	0.223
the group of 21-year-old undergraduate students	21 *years*	0.208
the group of 22-year-old undergraduate students	22 *years*	0.115
the group of older than 22-years-old undergraduate students	> 22 *years*	0.047

S4 Table. Groups by age. According to the age attribute of students, the node of the SVCN is divided into seven groups: < 18 *years*, 18 *years*, 19 *years*, 20 *years*, 21 *years*, 22 *years* and > 22 *years*. < 18 *years* denotes the group of under 18-year-old undergraduate students, 18 *years* denotes the group of 18-year-old undergraduate students, 19 *years* denotes the group of 19-year-old undergraduate students, 20 *years* denotes the group of 20-year-old undergraduate students, 21 *years* denotes the group of 21-year-old undergraduate students, 22 *years* denotes the group of 22-year-old undergraduate students and > 22 *years* denotes the group of older than 22-years-old undergraduate students. Followed by the fraction of seven groups in the SVCN respectively
