# Supplementary material for: Inferring and analysis of social networks using RFID check-in data in China
Source: PLoS One. 2017 Jun 1;12(6):e0178492. doi: 10.1371/journal.pone.0178492 (PMC5453530; doi:10.1371/journal.pone.0178492)
Supplement: S5 Table — According to the region attribute of students, the node of the SVCN is divided into three groups. We also consider Hong Kong, Macao and Taiwan, referred to as HMT. Finally,the fraction of EAST, CENTRAL, WEST and HMT in the SVCN respectively. (PDF) [file pone.0178492.s007.pdf]

full name of region group	abbreviation	the provinces, municipalities, Autonomous Regions and Special Administrative Region of this region containing	fraction
the eastern region of china	$EAST$	Beijing, Tianjin, Shanghai, Hebei Province, Jiangsu Province, Shandong Province, Liaoning Province, Zhejiang Province, Fujian Province, Guangdong Province and Hainan Province	0.203
the central region of china	$CENTRAL$	Heilongjiang Province, Jilin Province, Henan Province, Shanxi Province, Anhui Province, Jiangxi Province, Hubei Province and Hunan Province	0.564
the western region of china	$WEST$	Shaanxi Province, Sichuan Province, Gansu Province, Yunnan Province, Guizhou Province, Qinghai Province, Chongqing, Guangxi Zhuang Autonomous Region, Ningxia Hui Autonomous Region, The Tibet Autonomous Region, Xinjiang Uygur Autonomous Region and Inner Mongolia Autonomous Region	0.228
Hong Kong, Macao and Taiwan	HMT	Hong Kong, Macao and Taiwan	0.005

S5 Table. Groups by region. According to the region attribute of students, the node of the SVCN is divided into three groups: $EAST$, $CENTRAL$, and $WEST$. $EAST$ denotes the group of students from eastern region of china(relatively high level of economic development), $CENTRAL$ denotes the group of students from central region of china(middle level of economic development), $WEST$ denotes the group of students from western region of china(relatively low level of economic development). We also consider Hong Kong, Macao and Taiwan, referred to as HMT,which denotes the group of students from Hong Kong, Macao and Taiwan. Followed by the provinces, municipalities, Autonomous Regions and Special Administrative Region of every region consisting of. Finally,the fraction of $EAST$, $CENTRAL$, $WEST$ and HMT in the SVCN respectively.
